# CELLULAR, A Cell Autophagy Imaging Dataset

**DOI:** 10.1038/s41597-023-02687-x

**Published:** 2023-11-16

**Authors:** Amani Al Outa, Steven Hicks, Vajira Thambawita, Siri Andresen, Jorrit M. Enserink, Pål Halvorsen, Michael A. Riegler, Helene Knævelsrud

**Affiliations:** 1 Department of Molecular Medicine, Institute of Basic Medical Sciences, University of Oslo, Oslo, Norway; 2 Centre for Cancer Cell Reprogramming, Institute of Clinical Medicine, Faculty of Medicine, University of Oslo, Oslo, Norway; 3 Department of Molecular Cell Biology, Institute for Cancer Research, Oslo University Hospital, Oslo, Norway; 4 Simula Metropolitan Center for Digital Engineering, Oslo, Norway; 5 Section for Biochemistry and Molecular Biology, The Department of Biosciences, Faculty of Mathematics and Natural Sciences, University of Oslo, Oslo, Norway; 6 Oslo Metropolitan University, Oslo, Norway; 7 University of Oslo, Oslo, Norway; 8 UIT The Arctic University of Norway, Tromsø, Norway

## Abstract

Cells in living organisms are dynamic compartments that continuously respond to changes in their environment to maintain physiological homeostasis. While basal autophagy exists in cells to aid in the regular turnover of intracellular material, autophagy is also a critical cellular response to stress, such as nutritional depletion. Conversely, the deregulation of autophagy is linked to several diseases, such as cancer, and hence, autophagy constitutes a potential therapeutic target. Image analysis to follow autophagy in cells, especially on high-content screens, has proven to be a bottleneck. Machine learning (ML) algorithms have recently emerged as crucial in analyzing images to efficiently extract information, thus contributing to a better understanding of the questions at hand. This paper presents CELLULAR, an open dataset consisting of images of cells expressing the autophagy reporter mRFP-EGFP-Atg8a with cell-specific segmentation masks. Each cell is annotated into either basal autophagy, activated autophagy, or unknown. Furthermore, we introduce some preliminary experiments using the dataset that can be used as a baseline for future research.

## Background & Summary

**Fig. 1:**
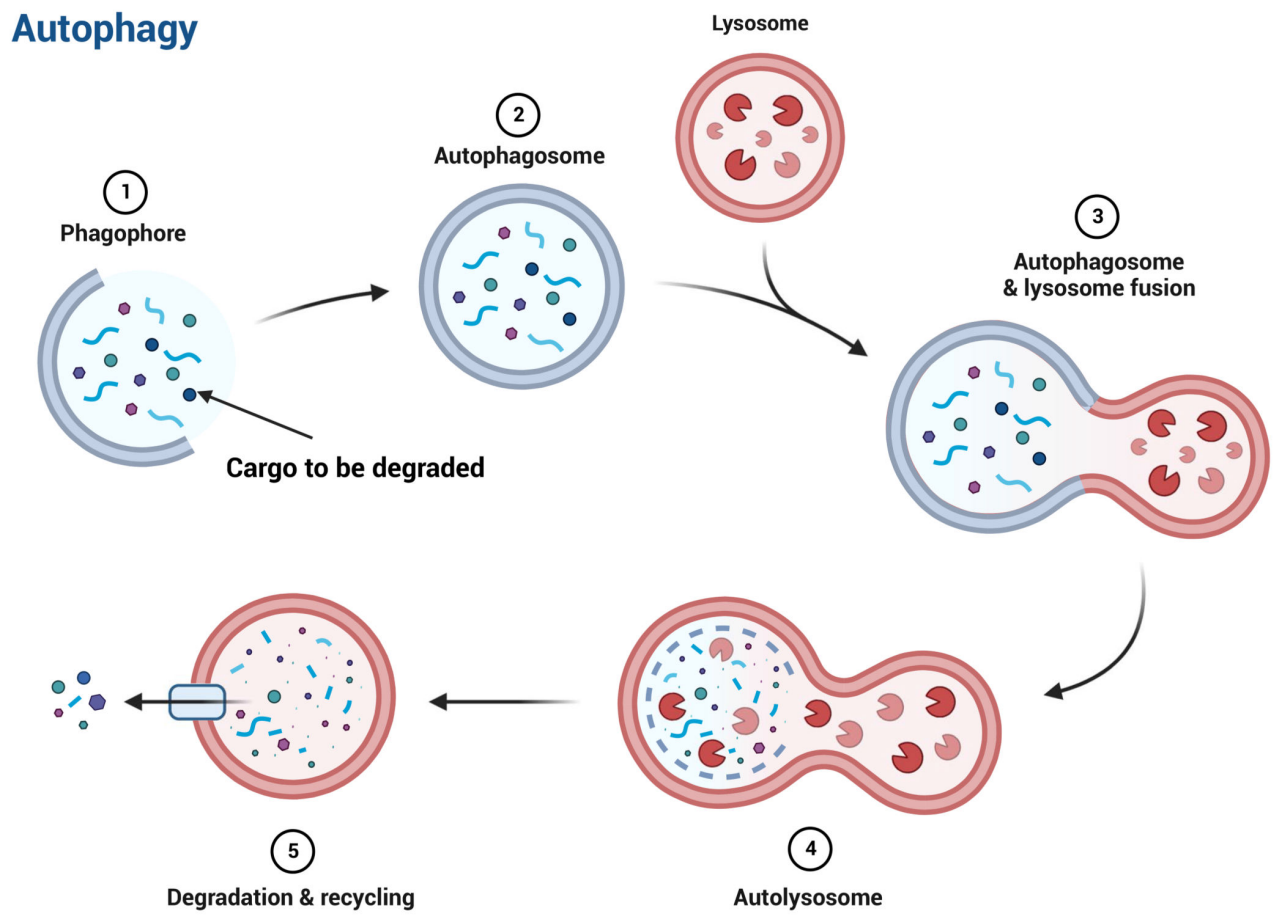
Schematic representation of key events during autophagy. Adapted from “Autophagy Process”, by BioRender.com (2022). Retrieved from https://app.biorender.com/biorendertemplates.

Autophagy is a highly conserved process that cells use to recycle damaged or unnecessary components. The autophagy process occurs inside eukaryotic cells and is crucial for cellular homeostasis. Autophagy delivers cytoplasmic material to membrane-bound organelles called lysosomes, thereby allowing the recycling of intracellular components^1^. “Autophagy” is derived from the Greek words auto-, means “self ”, and phagein, means “to eat”, thus denoting “self-eating”. There are three main types of autophagy: macroautophagy, microautophagy, and chaperone-mediated autophagy^2^. In this paper, we focus on macroautophagy (hereafter referred to as autophagy). Briefly, autophagy begins with a series of molecular events leading up to the generation of double-membrane vesicles called autophagosomes, which contain the cargo to be degraded. The autophagosomes later fuse with lysosomes, forming autolysosomes where degradation of cargo takes place and the resulting building blocks are recycled back to the cytoplasm (Fig. 1).

Autophagy exists at basal and activated levels. Basal autophagy involves regular recycling of superfluous or impaired cellular material under nutrient-rich conditions^3^ and therefore secures cellular homeostasis^4^. On the other hand, stressful cellular events such as changes in nutritional status^5^ activate higher levels of autophagy as a pro-survival response. During starvation, the degradation of cellular material in autolysosomes ensures the availability of building blocks that are reutilized for the synthesis of macromolecules^1^.

Autophagy is initiated by a set of conserved autophagy-related proteins (Atg- proteins). Several multi-subunit complexes, such as the Atg1/ULK1 complex, the Vps34 complex I, and the two ubiquitin-like proteins Atg8/LC3 and Atg12, along with their conjugation systems are involved in the nucleation and expansion of autophagic membranes^1^. Autophagy can be monitored by fluorescently labeled proteins of the ubiquitin-like Atg8 family^6,7^. The cellular route of Atg8 proteins facilitate monitoring the autophagic process as Atg8 originally exists in the cytosol, but upon induction of autophagy it gets conjugated to the lipid phosphatidylethanolamine (PE)^8^. Atg8–PE localizes to both the inner and outer membranes of the formed autophagosomes, whereby Atg-8-PE on the inner membrane will be delivered to the lysosome where it undergoes degradation^9^.

Autophagic lysosomal degradation is essential for maintaining cellular homeostasis, and its deregulation is associated with several diseases such as cancer, neurodegeneration, and inflammation^10^. Thus, autophagy constitutes a potential target for therapy. This creates a demand for a deeper understanding of the autophagic landscape in a cell to gain better control over the therapeutic modulation of the pathway. The investigation of autophagy using different autophagic assays, especially on large-scale screens, has helped researchers uncover basic mechanisms and speed up therapeutic discovery^11^. Automated image processing and analysis when following autophagy states of cells is often seen as one of the throughput bottlenecks, especially in large-scale screens, and could hamper the complete grasping of the different phenotypes at hand. Recently, machine learning (ML) has contributed to different fields of research such as medicine^12^, finance^13^, and biomedical engineering^14^. In ML, preset tasks are executed by the use of algorithms that learn from labeled or unlabeled data. Basic ML tasks can be regression, classification, detection, segmentation, and generation tasks. One of the tasks that ML has been instrumental in advancing is image analysis, and ML has already shown potential in image analysis of autophagy in cells. For example, Zhang *et al*.^15^ presented a deep learning framework to quantitatively measure autophagic activity in yeast cells. The results demonstrated that deep learning approaches are promising in the analysis of different types of autophagic phenotypes. However, ML requires a lot of data to work well, and public annotated samples of autophagy-related images are scarce.

**Fig. 2:**
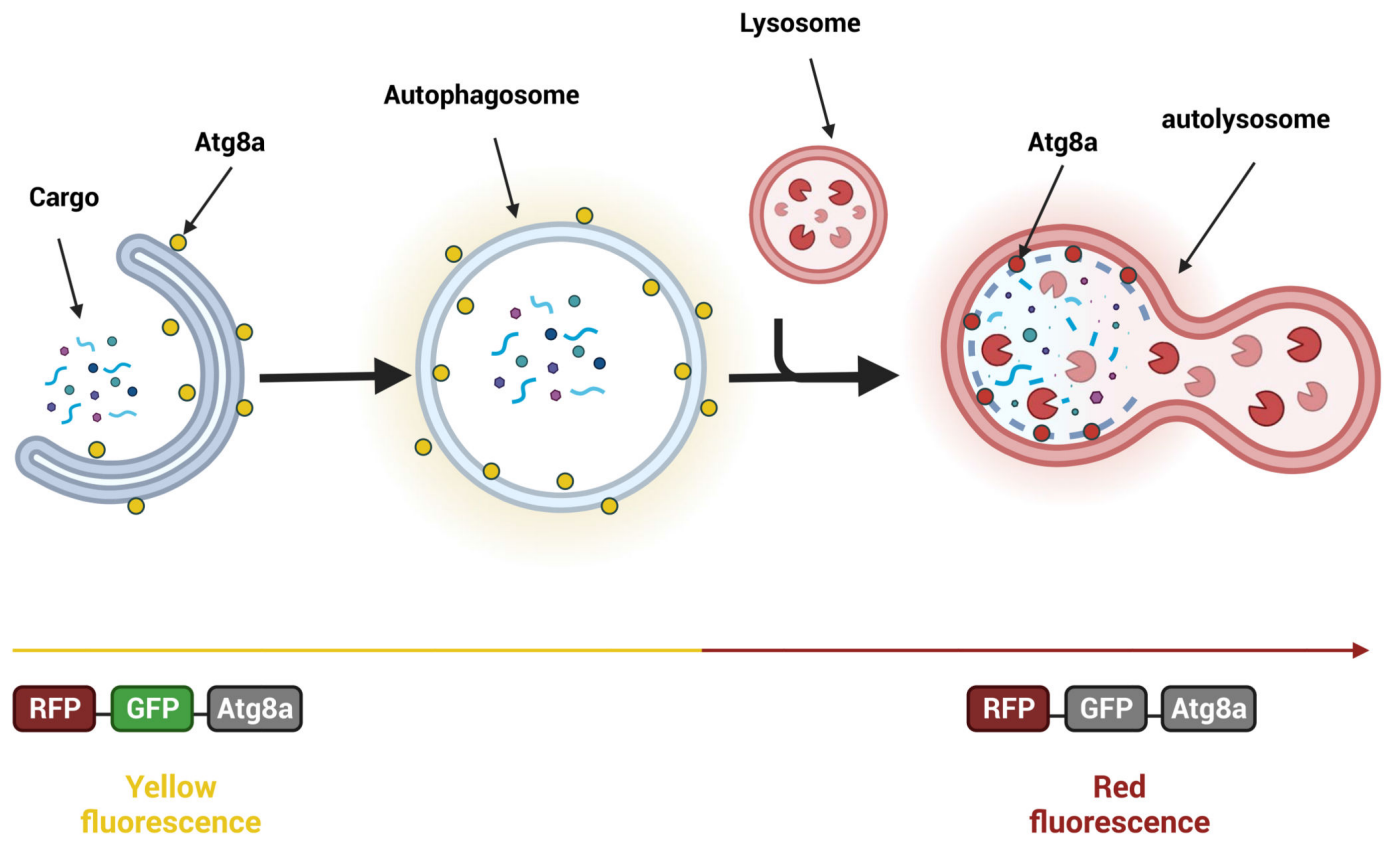
Schematic representation of the autophagic flux assay. A tandem fusion of mRFP and EGFP is fused to Atg8a to form a pH-sensitive sensor used to monitor autophagy flux in live cells. The RFP tag is acid-insensitive, while the GFP tag is acid-sensitive. Both tags emit fluorescent light in autophagosomes resulting in overlapping green and red fluorescence (“yellow”). The fusion of autophagosomes with lysosomes results in formation of acidic autolysosomes where the green fluorescence from GFP is lost (quenched). The red fluorescence from RFP is more stable, resulting in red fluorescence only. (Created with Biorender.com).

In this paper, we present a dataset containing time-lapse live-cell imaging data of *Drosophila* S2 cells stably expressing the tandem-tag mRFP-EGFP-Atg8a reporter^16^ in both basal and activated autophagy states. The tandem tag is a popular and frequently used tool both *in vivo* (in model organisms) and *in vitro* (in cell culture) in the field of autophagy because it monitors flux through the autophagy pathway (see Fig. 2). Furthermore, it also allows for the visualization of all autophagic structures. Autophagosomes will be identified as spots with combined green and red fluorescence that appear as yellow dots in merged fluorescence images. However, upon induction of autophagy, such as upon stresses like starvation, the tandem-tag Atg8 reporter will be delivered to autolysosomes where the GFP fluorescence is quenched due to low pH. As a result, autolysosomes will be visible as red fluorescent spots lacking green fluorescence^17^. Since autophagy is conserved from yeast to man, discoveries in *Drosophila* S2 cells will likely be applicable across species, including in a human disease context.

The main contributions of this paper are as follows:

1. A public and free-to-use dataset containing 18,720 microscopic images of Drosophila melanogaster S2 cells. Of the 18,720 files, 159 (53 images with three wavelengths) have been labeled with cell-specific segmentation masks and bounding boxes, and each cell is classified into basal or activated autophagy.
2. A set of preliminary baseline experiments meant to provide a benchmark for future researchers.

The remainder of this paper is structured as follows. First, we give an overview of how the data were collected and annotated. Then we detail how the dataset is structured, including the folder structure and information about the files contained within. Next, we describe a set of example use case scenarios that may be supported by using the presented dataset. Furthermore, we suggest metrics that should be used when evaluating the performance of a ML model when executing the example scenarios described above. We then present benchmark experiments meant to be a starting point for future researchers. Finally, we conclude this paper with a brief discussion of the applications of this dataset.

## Materials and Methods

In this section, we describe how we collected the images of the cells and how we performed the annotations.

### Preparation of cultured cells

*Drosophila* S2-mRFP-EGFP-Atg8a cells^16^ were grown according to standard procedures in ESF921 medium (Expression Systems, 500302) in the presence of 0.01 mg/mL of Zeocin as a selection reagent (Gibco, R25001) in a 25°*C* incubator. Experiments were performed three days after cell passage and repeated twice. Around 80,000 cells in 50 μL of ESF921 media were seeded per well of 384-well glass bottom plate with a black frame (Cellvis, P384-1.5H-N). The cells were left to grow in a 25 °*C* incubator overnight. On the second day, around 20 μL of cell suspension was transferred from each well to its corresponding well in a new 384-well glass bottom plate coated with 0.5 mg/mL concanavalin A (Sigma, L7647) to increase cell attachment. The volume was completed to 50 μL with IPL-41 insect media (Sigma, I7760). This media was used as the nutrient-rich media during imaging since it exhibits less autofluorescence than ESF921. Cells were kept for around two hours in the new media before imaging.

### High-content live-cell imaging

To image the cells under nutrient-rich and amino-acid-deprived conditions, a state-of-the-art setup was used. Time-lapse live-cell imaging was performed at room temperature using an ImageXpress Micro Confocal High-Content Imaging System Microscope from Molecular Devices. Wide field acquisition mode was used, and three different images from three different visiting points were taken from each well with a 40× Plan Fluor ELWD air objective lens to capture green fluorescence (GFP), red fluorescence (RFP), and transmitted light (TL) signals. Before imaging, cells were washed once with either IPL-41 (Sigma) as a nutrient-rich medium (fed) or with Live-cell imaging solution (Molecular probes, A14291DJ) supplemented with 2 mg/mL glucose (Formedium, GLU03) (abbreviated as LCMG) as an amino-acid-deprived medium (to induce stress by starvation; starved). Cells were imaged for four hours with one-hour intervals either in IPL-41 for a fed condition or in LCMG for a starved condition.

### Flow cytometry

EGFP and mRFP expression levels were assessed in fed mRFP-EGFP-Atg8a S2 cells by flow cytometry. Cells were centrifuged at 500 g for 5 minutes and resuspended in 1 mL IPL-41 media, centrifuged again and then resuspended in 1 mL IPL-41 media. Hoechst (Hoechst 33258, Thermo Fisher, H3569) was added at a final concentration of 0.008 μg/mL at room temperature to enable exclusion of dead cells during analysis. Samples were then filtered through 35 mm filter cap tubes (Falcon, 352235) and ran through LSR II (BD) flow cytometer. Laser 488 nm with bandpass filter 525/30 nm and longpass Dichroic filter 505 nm was used to detect EGFP, and laser 561 nm with bandpass filter 582/15 nm and longpass Dichroic filter 570 nm was used to detect mRFP. Around 20,000 events (cells) were used for the analysis using the software FlowJo v10 (BD). S2 cells (DGRC) with no reporter expression were used as a negative control.

### Cell annotation

**Fig. 3:**
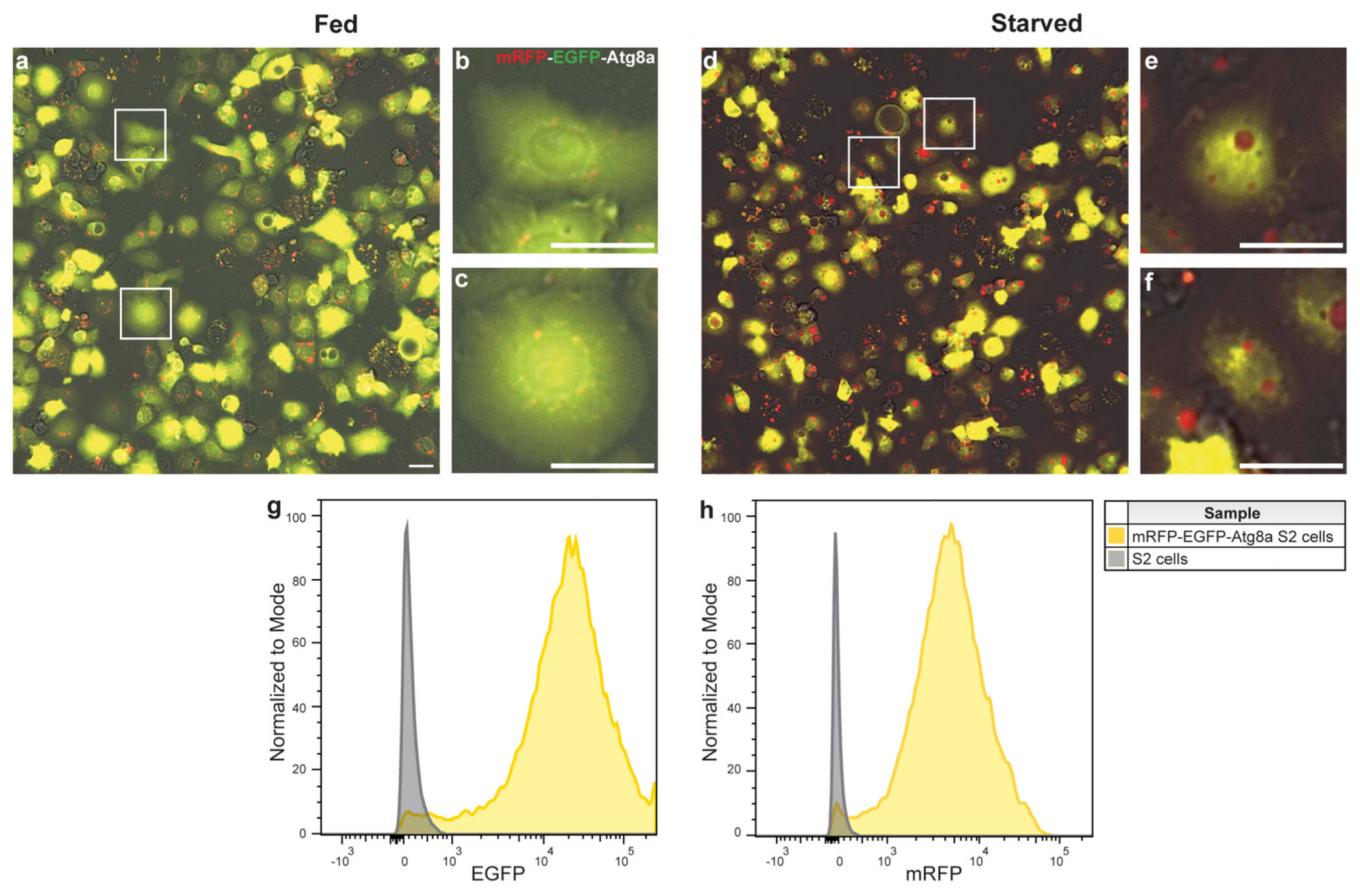
Representative images of S2 cells expressing mRFP-EGFP-Atg8a after four hours of either a fed condition (**a**–**c**) or a starved condition (**d**–**f**). (**b,c**) and (**e,f**) represent magnified cells in areas indicated by boxes in (**a**) and (**d**) respectively. Scale bars = 20 μm. Fed mRFP-EGFP-Atg8a S2 cells were subjected to flow cytometry to detect cells with green (**g**) or red fluorescence (**h**). S2 cells with no reporter expression were used as negative control.

Cells were annotated using LabelBox^18^ into three separate classes in merged colored images, namely, basal autophagy, activated autophagy, and unknown. For this purpose, the three channels (GFP, RFP, and TL) images per visiting point per well were merged into one colored *JPG* image with the GFP channel given green color, the RFP channel given red color, and the TL channel given grey color. Brightness and contrast were adjusted equally across the whole image using image J software^19^ (the resulting images are hereafter referred to as the merged colored images). Figure 3 shows examples of cells from a fed (basal autophagy) and stressed/ starved (activated autophagy) condition. mRFP-EGFP-Atg8a spots are predominantly small and show overlap between green and red (yellow in color) in a fed state, whereas spots become red and much bigger upon stresses like starvation due to autophagy activation over time. For annotation purposes, cells with basal autophagy were identified as cells with small spots that were mainly yellow in color, while cells with activated autophagy were identified as cells with many small or big red spots. Unknown cells are cells that did not fall into the aforementioned classes. To analyze whether this unknown class could be explained by a population of cells that did not express the mRFP-EGFP-Atg8a reporter even under continued antibiotic exposure, the fluorescence intensity of mRFP-EGFP-Atg8a S2 cells in a fed condition was analyzed by flow cytometry. Figure 3g,h shows that there was a small population (2%–4%) of cells that overlapped with the negative control. The apparent absence of reporter expression in this population obviously prevents classification. However, the major reasons for annotation as “unknown” were: very high or very low expression of the green/red fluorescence reporter, out-of-focus cells, highly aberrant cell shape or clumped cells. In all of these cases, the autophagy state could not be determined because the green/red spots could not be properly distinguished or evaluated.

**Fig. 4:**
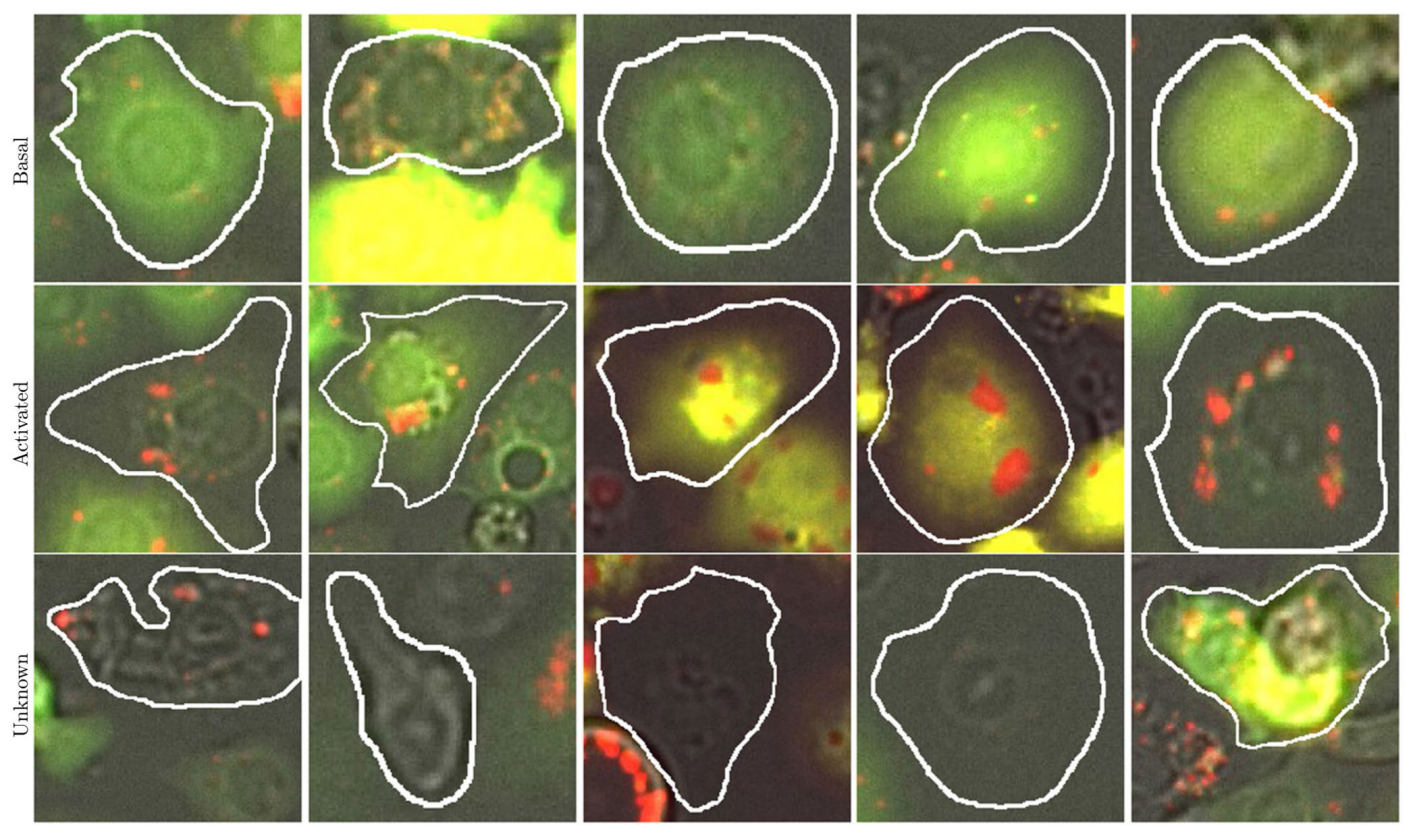
Examples taken from the three classes provided in the dataset. The objects of interest are outlined in white.

Three different scientists in the field of autophagy annotated all of the cells presented here on the basis of the criteria mentioned above. Before beginning the annotation, the scientists met and discussed some test images to agree on the criteria to be followed for classifying the cells. During the process of annotation, follow-up meetings were arranged when the annotators encountered cells they did not know how to classify, or when the scientist reviewing the annotations detected deviations from the agreed-upon criteria. 53 images in total from either a nutrient-rich medium (fed) or amino-acid deprived medium (stressed/starved) were mainly annotated by two scientists who were blinded to the conditions of the image and the majority of annotations were reviewed by a third scientist. Although this manual annotation is very time-consuming, challenging, and subject to uncertainty, by imposing our quality control measures described above, we have sought to minimise uncertainty and bias. Figure 4 shows some example cells taken from each of the classes mentioned and the corresponding manually annotated ground truth.

## Data Records

CELLULAR is available on Zenodo (https://zenodo.org/record/8315423)^20^ and is licenced under Creative Commons Attribution 4.0 International (CC BY 4.0). The dataset contains various data types that are useful for different applications. In addition to the raw images collected by microscopy, the dataset also includes the merged colored images that were used for annotation and analysis. The dataset also includes segmentation masks in *PNG* format for each cell along with cell-specific classifications. Each mask is stored in a separate file, which is stored in a directory corresponding to its classification. Additionally, the dataset contains bounding box coordinates for each cell, along with cell-specific classifications. Overall, the dataset contains 18,720. *TIF* files, corresponding to 6,240 image samples, for which there is also a merged colored image that was used to perform the annotations. Of these 6,240 samples, 53 were annotated with cell-specific annotation masks and bounding boxes. The resolution of each image is 2048 × 2048 and contains between 140 and 386 annotated cells (bounding boxes and segmentation masks). Overall, the dataset contains 4,539 cells classified in the basal autophagy state, 3,191 cells in the activated autophagy state, and 6,294 cells classified as unknown. An overview is also given in Table 1.

The dataset contains raw microscope images stored as three separate TIF files, with each file representing a single channel: green (w1), red (w2), and transmitted light (w3). Each file has a name that indicates a specific experiment, well position in the 384-well plate, and visiting point within each well. Three visiting points were imaged per well (s1, s2, and s3). The file naming format is: Timepoint_<timepoint_id><experiment_date>-ST<well><visiting_point><wavelength>.timepoint_id is the timestamp for the timelapse video, ranging from 1 to 5, with 1-hour intervals between images over a 4-hour duration. experiment_date identifies the date on which the experiment was performed. well signifies the well from which the image is collected from, and visiting_point is the visiting point within that well. Wells are assigned specific letters ranging from C to N. wavelength denotes the image wavelength type. In one experiment (date: 220518), wells C, D, E, F, G, and H were fed, while wells I, J, K, L, M, and N were starved. In another experiment (date: 220524), wells C, D, and E were fed, while wells F, G, H, I, J, K, L, and M were starved. A well-established cell line of *Drosophila* S2 cells was used to generate the data presented here. No ethical approval is required for the use of such cell lines.

**Fig. 5:**
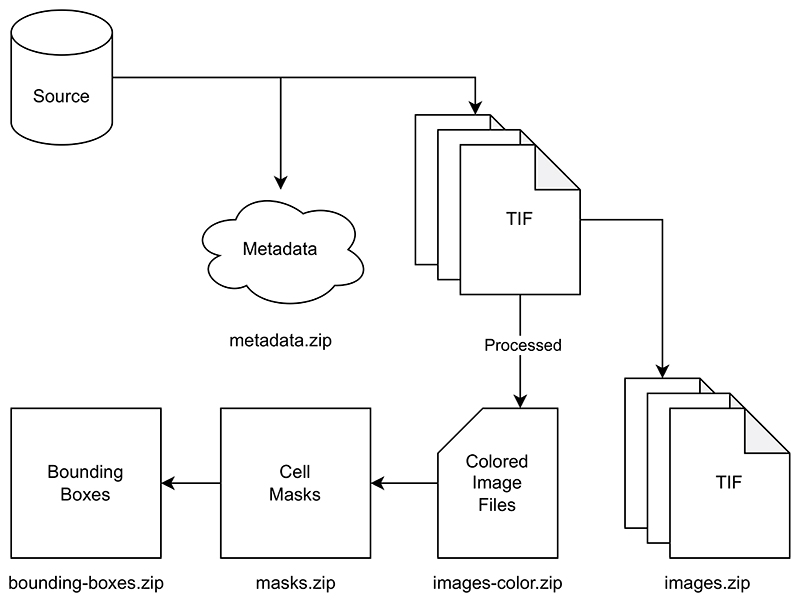
An illustration of how the dataset is prepared and structured in Zenodo. The TIF files are extracted from the source database and then used to produce colored images. The colored images are used to produce the segmentation masks, which are done on a cell-based level. The bounding boxes are created from the segmentation masks based on the vertical and horizontal boundaries. Additional metadata is extracted from the source database and included in the dataset.

The TIF files are provided in the *images.zip* directory, consisting of three files per image as previously described. Merged color images are provided as JPG files in the *images-color.zip* directory. Segmentation masks are stored as PNG files in the *masks.zip*. Bounding box coordinates follow the YOLOv5 format and are stored as TXT files in the *bounding-boxes.zip* directory. Additional metadata about the microscope configuration is provided in the *metadata.zip* directory. A visualized structure of the dataset is shown in Fig. 5. The dataset is available online and licensed under the Creative Commons Attribution 4.0 International (CC BY) license.

## Technical Validation

To evaluate the technical quality of CELLULAR, we performed some simple baseline experiments using current state-of-the-art methods within cell segmentation. We use the Cellpose^21^ framework to train and evaluate models to detect and segment cells. As mentioned before, this provides a technical evaluation of the data in addition to a baseline that can be a starting point for future research using it. We tested four different models, (i) a out-of-the-box pre-trained cyto model (ii) a out-of-the-box pre-trained cyto2 model, (iii) a fine-tuned cyto model, and (iv) a fine-tuned cyto2 model. All experiments were performed on a machine running Arch Linux with an Intel Core i9-10900k, 32 gigabytes of RAM, and an RTX 3090 graphics card. The experiments were implemented in Python using the PyTorch library^22^.

We trained each model using the default configuration provided by Cellpose, with a few objections. All fine-tuned models were trained for 500 epochs with a learning rate of 0.2 and a batch size of eight. The models are trained to predict cells independently, distinguishing one cell from another. This is done by training on the cell-specific masks included in the *masks* directory of the dataset and follows the training procedure explained in Cellpose^23^. The exact configuration used is available on our GitHub repository, including scripts to reproduce the experiments. The weights of the trained models are also included.

**Fig. 6:**
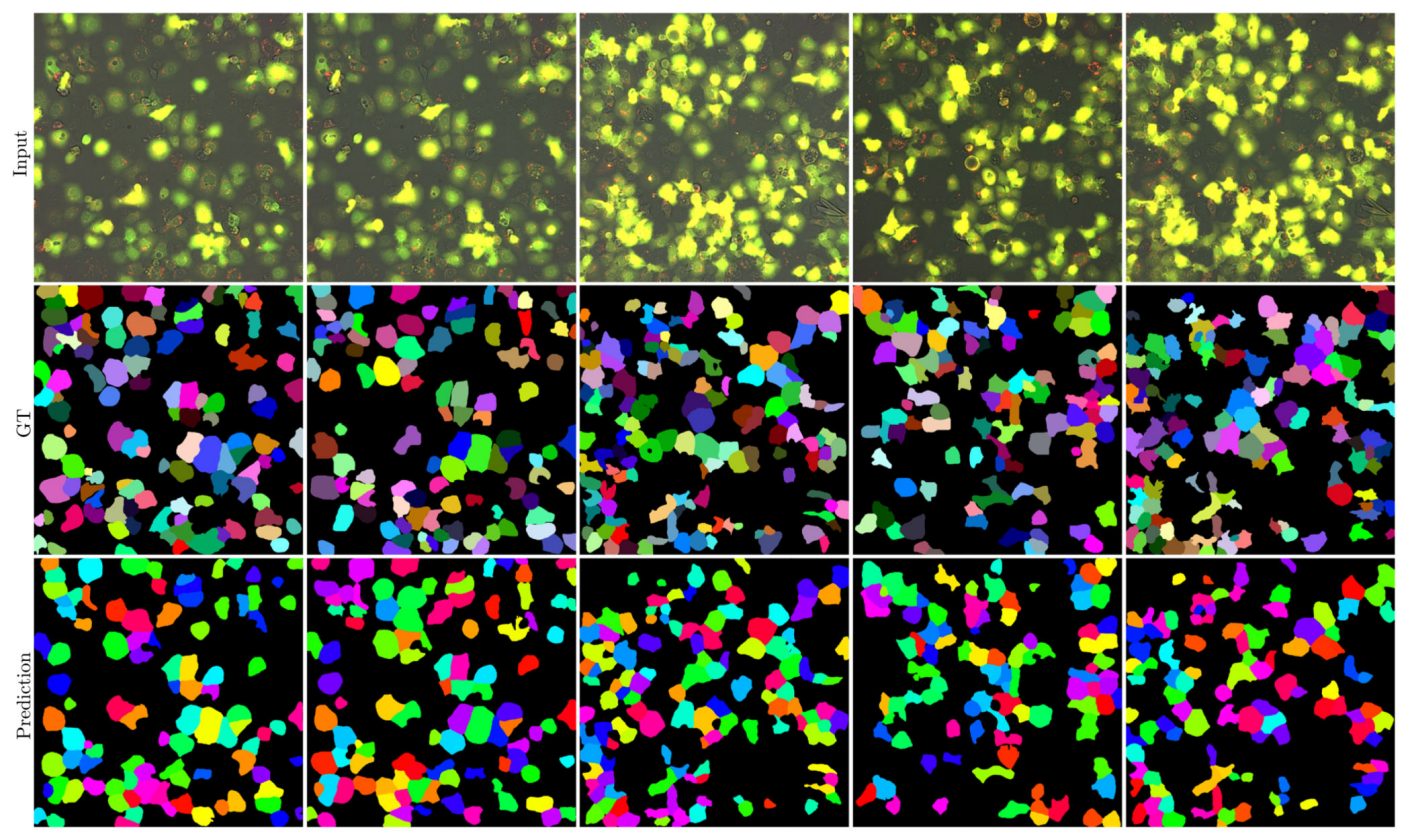
A visualization of the Cellpose segmentation results from the fine-tuned cyto2 model. The first row shows the input image, the second row shows the ground truth, and the third row shows the predicted masks. Each column represents a different timepoint. Note that the colors are used to differentiate between the different cells.

Given the results shown in Table 2 and Fig. 6, we can see that the dataset can be used to address different interesting challenges in the field of autophagy, but that there is still room for improvement over the provided baseline models. Finally, the purpose of the presented experiments has two main goals. First, we wanted to provide a baseline for future experiments using the dataset that is easy to reproduce.

Second, we demonstrate the challenging nature of the problem, indicating that further research is needed to enhance the quality of the algorithm and develop task-specific algorithms utilising the given data set. For instance, the out-of-the-box Cellpose model, often used as standard method in this type of analysis, proves insufficient.

## Usage Notes

The main domain of application of this dataset is training machine learning (ML) models (supervised, unsupervised, semi- or self-supervised) to automatically detect and segment cells in microscopy images and determine whether the detected cells are in basal or activated autophagy. Its applications extend beyond autophagy detection, contributing to broader areas of cellular analysis and enhancing the scientific understanding of cellular structures and functions. For optimal results, the models trained on this dataset should be applied to data collected under similar conditions. Here are some of the key use cases:

- **(i) Cell Detection and Segmentation**. Microscopy images can be used to detect and segment cells, enabling a deeper analysis of cell type, shape, and other attributes. This plays a crucial role in understanding cell morphology and its implications in health and disease.
- **(ii) Autophagy State Analysis**. The dataset allows for an analysis of the autophagy state of an individual cell, contributing to research on cell stress, cell growth, and survival mechanisms.
- **(iii) Cell time-specific analysis**. Each sample contains images taken at different time points, allowing the tracking of specific cells over time. This could be an interesting point of analysis for future studies.
- **(iv) Microscopy Image Transformation**. The raw microscopy images of cells can be automatically converted into more interpretable images, aiding in efficient and effective visual inspections.
- **(v) Autophagy Mechanism Simulation**. The dataset can be employed to simulate autophagy mechanisms, promoting an enhanced understanding of these processes and supporting the generation of more data for future studies.
- **(vi) Exploration of Self- or Semi-Supervised Learning Problems**. For example, the problem of learning by counting can be approached using this dataset, supporting the development of models capable of counting cells or other structures in microscopy images.
- **(vii) Multi-Modal Data Integration**. This dataset could be leveraged in a multi-modal data integration context, where information from other datasets such as genomic or proteomic data could be combined with the microscopy images to provide a more comprehensive analysis.
- **(viii) Computer Vision**. The dataset could be used to develop new computer vision algorithms specifically designed for the application. But also development of more general methods and the usage of the data in benchmarks and competitions are interesting areas to explore.

The applications of this dataset extend across a range of cellular analysis domains, from enhancing autophagy detection and segmentation to supporting multi-modal data integration and computer vision. It opens up numerous opportunities for leveraging machine learning in cell biology, paving the way for more comprehensive and nuanced studies. By facilitating the transformation of raw microscopy images into interpretable visual data, enabling autophagy mechanism simulations, and exploring self- or semi-supervised learning problems, this dataset stands to make significant contributions to the field of cellular analysis and beyond.

### Suggested metrics

Depending on the application, a different set of metrics should be used to adequately measure the performance of a model for a given task. The appropriate metrics will depend on the specific application of the model and will help to provide a complete picture of its performance. CELLULAR offers support for several different types of ground truth, including classification, detection, and segmentation. For classification tasks, we recommend using standard multiclass classification metrics such as precision, recall, F1-score, and Matthews correlation coefficient (MCC). These metrics can help to determine the accuracy and reliability of the model’s predictions. For segmentation tasks, metrics such as the Dice coefficient and the intersection over union (IoU) are recommended. These metrics can help to evaluate the model’s ability to accurately segment objects in images. For detection tasks, average precision and mean average precision are appropriate metrics to use. These metrics can help to determine the model’s ability to accurately locate and identify cells in the images. No matter what the task is, it is always important to use several metrics in order to provide a comprehensive view of a model’s performance.

## Supplementary Material

Supplementary file 1 The online version contains supplementary material available at https://doi.org/10.1038/s41597-023-02687-x.

Supplementary file 2

## Figures and Tables

**Table 1:** An overview of how many samples of each cell class are in the dataset.

Class	# Samples
Basal	4,539
Activated	3,191
Unknown	6,294
Total	14,024

**Table 2:** Results of baseline Cellpose models on the testing dataset for cell segmentation.

Model	Class	Precision	Recall	F1-Score	Jaccard
cyto	Fine-Tuned	0.8210	0.7575	0.7865	0.6496
	Pre-Trained	0.4469	0.3630	0.3982	0.2503
cyto2	Fine-Tuned	0.8153	0.7614	0.7864	0.6491
	Pre-Trained	0.5486	0.6572	0.5967	0.4280

## Data Availability

The code and models used to perform the experiments are available online at the following link: https://github.com/simula/cellular.
